# Genome-Wide Identification and Phylogenetic Characterization of the FTIP Gene Family in Maize (*Zea mays*)

**DOI:** 10.3390/genes16050539

**Published:** 2025-04-30

**Authors:** Guihua Lv, Fangjian Li, Jianjian Chen, Zhenxing Wu, Tingzhen Wang, Haiping Ding, Zhiming Zhang, Fazhan Qiu

**Affiliations:** 1 Hubei Hongshan Laboratory, National Key Laboratory of Crop Genetic Improvement, Huazhong Agricultural University, Wuhan 430070, China; lvgh@zaas.ac.cn (G.L.); dinghp@sdau.edu.cn (H.D.); 2 Zhejiang Academy of Agricultural Sciences, Institute of Maize and Featured Upland Crops, Hangzhou 310015, China; lifangjian@zaas.ac.cn (F.L.); chenjj@zaas.ac.cn (J.C.); wuzx@zaas.ac.cn (Z.W.); wangtz@zaas.ac.cn (T.W.); 3 National Key Laboratory of Wheat Breeding, College of Life Sciences, Shandong Agricultural University, Taian 271018, China

**Keywords:** *ZmFTIP*, *ZmFTIP*-like, MCTPs, phylogenetic analysis, maize

## Abstract

The maize FT-interacting protein (*FTIP*) gene family represents a group of multiple C2 domain and transmembrane proteins (MCTPs), characterized by their unique structural motifs and membrane-spanning regions., plays crucial roles in intercellular communication and stress responses. Here, we systematically characterized 27 *ZmFTIP* genes unevenly distributed across 10 maize chromosomes. Phylogenetic analysis with rice, soybean, and Arabidopsis homologs revealed five evolutionary clades with monocot-specific conservation patterns. Promoter cis-element profiling identified hormone-responsive (ABA, JA, auxin) and stress-related motifs, corroborated by differential expression under abiotic stresses and phytohormone treatments. Notably, *ZmFTIP18* and *ZmFTIP25* showed sustained upregulation under cadmium exposure, while *ZmFTIP13* exhibited downregulation. Synteny analysis demonstrated strong conservation with monocot *FTIP*s, suggesting ancient evolutionary origins. This comprehensive study provides foundational insights into *ZmFTIP* functional diversification and potential biotechnological applications.

## 1. Introduction

Plants regulate the position and developmental information of plant cell division and differentiation by transducing signals through receptor-like kinases or transmitting transcription factors [1]. This communication is essential for plant growth and development to respond to internal and external signals and is regulated by intracellular and extracellular transport pathways [2]. In order to conduct signal transduction more smoothly, plants have evolved a complex endomembrane system that effectively separates signal molecules and promotes the transport of protein and RNA macromolecules between endomembrane compartments [3].

Recent studies have found that highly conserved multi-C2 domain and transmembrane region proteins (*MCTP*s) are key signal molecules that mediate the transport of regulatory substances in plant cells [4]. These proteins contain 3–4 C2 domains at the N-terminus and 1– 4 transmembrane regions at the C-terminus. As a eukaryotic lipid-binding domain, the C2 domain can target the intracellular membrane [5]. In membrane transporters, different C2 domains carry unique conserved sequences and achieve specific cellular functions in a synergistic manner rather than simply superimposed [6].

In the genome of *Arabidopsis thaliana*, 16 *MCTP* genes have been identified, of which two members, QUIRKY (QKY) and FT-interacting protein 1 (*AtFTIP1*), have been shown to be involved in the transport of macromolecules [7,8,9]. Specifically, QKY regulates plant organ development by interacting with the leucine-rich repeat-containing receptor-like kinase STRUBBELIG (SUB) [9]. On the other hand, *AtFTIP1* is anchored on the endoplasmic reticulum (ER) membrane and participates in the transport of florigen FLOWERINGLOCUST (FT) from companion cells to sieve tube cells, thereby regulating the flowering timing of *Arabidopsis thaliana* [8]. *AtMCTP3*, *AtMCTP4*, and *AtMCTP6* control auxin response factors (ARFs) to determine lateral root development [10]. *AtFTIP3* and *AtFTIP4* prevent the intracellular trafficking of the key regulator SHOOTMERISTEMLESS (STM) from peripheral shoot meristem region cells to the PM and play an important role in mediating Arabidopsis shoot stem cell proliferation and differentiation [11]. In rice (*Oryza sativa*), 13 *MCTP* genes have been identified [12]; *OsFTIP1*, an ortholog of *AtFTIP1*, regulates rice flowering time by regulating the transport of rice florigen RICEFLOWERINGLOCUST1 from companion cells to sieve tubes [13]; *OsFTIP7* is a factor in auxin-mediated anther dehiscence [14]. In addition, abscisic acid and jasmonic acid hormone treatment changed the transcription level of *OsFTIP* in rice [12].

Under drought stress conditions, the expression of *OsFTIP1* is downregulated, allowing *OsMFT1* to translocate into the nucleus. There, it modulates the expression of drought-resistance genes by regulating *OsMYB26* (a negative regulator) and *OsbZIP66* (a positive regulator) [15]. Using virus-induced gene silencing (VIGS) system to silence *GhMCTP* gene expression in cotton, *GhMCTP7*, *GhMCTP12*, and *GhMCTP17* were found to play important roles in bud meristem development [16].

Previous studies have identified 17 members of the MCIP gene family in maize [17]. A carbohydrate partitioning defective 33 (*cpd33*) mutant has been identified. This gene encodes a protein containing multiple C2 domains and transmembrane regions, which results in excessive carbohydrate accumulation in leaves. Its potential function might involve facilitating the symplastic movement of sucrose within the phloem [18]. In summary, members of the maize FTIP gene family may play a role in regulating maize flowering and coping with various biotic and abiotic stresses. However, the identification, evolution, expression pattern, and function of maize FTIP gene family members are not completely clear.

In maize research, 27 *FTIP* family members (designated as *ZmFTIP*s) were characterized through genome-wide screening. Subsequent investigations encompassed multi-dimensional analyses including gene architecture profiling, physical mapping across chromosomes, evolutionary motif conservation assessments, cis-element identification in upstream regulatory sequences, and phylogenetic tree construction for subfamily classification.The expression profiles of *ZmFTIP* genes in different organs and under different hormone treatments and stress conditions were studied. Synteny analysis of *ZmFTIP*s and their homologs in rice, Brachypodium, sorghum, barley, soybean, and Arabidopsis. In addition, this study elucidated the expression patterns of *ZmFTIP*s in maize under various stress conditions, thereby providing theoretical foundations for the potential application of *ZmFTIP*s in maize improvement.

## 2. Materials and Methods

### 2.1. Bioinformatics Analysis of ZmFTIP Family Members

The protein sequences of maize *FTIP*s were retrieved from the maizeGDB database (*Zea mays* RefGen_V5). (https://www.maizegdb.org/, accessed on 5 October 2023). Moreover, 27 maize FTIP family members were initially screened using integrated BLAST 2.14.0+, PFM, and motif-based methods. The ExPasy tool (https://web.expasy.org/protparam/, accessed on 5 October 2023) was utilized to predict the physicochemical properties of the ZmFTIP family, including molecular weight, theoretical isoelectric point, hydrophilicity index, and instability index. The subcellular localization of the *ZmFTIP* family members was predicted using Cell-PLoc 2.0, a web-based tool available at http://www.csbio.sjtu.edu.cn/bioinf/plant-multi/, accessed on 5 October 2023 [19]. *ZmFTIP*s were designated according to their physical locations on the maize chromosome.

### 2.2. Reconstruction of the Phylogenetic Tree for ZmFTIPs in Maize 

The amino acid sequences of *ZmFTIP*s were obtained from maize (*Zea mays* RefGen_V5). After blast treatment of soybeans (*Wm82.a4.* v6), Arabidopsis (*Araport11*), rice (*Oryza sativa* v7.0), Brachypodium (*B. distachyon* v3.2), barley (*H. vulgare* r1), poplar (*P. trichocarpa* v4.1), tomato (*S. lycopersicum* ITAG4.0), potato *(S.tuberosum* v6.1), wheat (*T. aestivum* v2.2), grape (*V. vinifera* v2.1), the sequences of *FTIP* family members were retrieved from Phytozome (https://phytozome-next.jgi.doe.gov/, accessed on 5 October 2023). The phylogenetic tree was reconstructed using the MEGA 7.0 (https://www.megasoftware.net, accessed on 5 October 2023) program using the maximum likelihood method with 1000 bootstrap replicates. Bootstrap values are indicated as percentages of major branches [20].

The conserved ZmFTIP motifs were identified and analyzed using the MEME suite (https://meme-suite.org/meme/tools/meme, accessed on 5 October 2023). Additionally, the conserved domains of the ZmFTIP family were characterized through the Batch CD Search tool available on the NCBI website (https://www.ncbi.nlm.nih.gov/Structure/bwrpsb/bwrpsb.cgi, accessed on 5 October 2023).

### 2.3. Plant Materials and Growth

The Zea mays genotype REFERENCE B73 (B73) was employed for this study. Maize seeds were surface-sterilized using a solution of sodium hypochlorite (100 mL NaClO supplemented with 4.2 mL of 36–38% HCl) for 4 h. The sterilized seeds were then evenly sown in clean, moist quartz sand and cultivated in a controlled growth chamber maintained at 25 °C under a photoperiod of 16 h light/8 h dark for 4 days. Uniformly developed seedlings were subsequently transplanted into a nutrient solution [21]. The nutrient solution was changed every 5 d. Maize seedlings were grown in a hydroponic device for 10 days (d), then the seedlings were treated with25 μM CdCl_2_, and samples were taken for measurement at 2 h (h) and 24 h, respectively. An appropriate number of roots and leaves were put into liquid nitrogen and quickly stored at −80 °C.

### 2.4. Cis-Regulatory Element Analysis of ZmFTIPs

For the analysis of cis-regulatory elements, a 2-kb region upstream of the transcription start site was utilized to predict cis-elements in promoter regions using the PlantCARE database (http://bioinformatics.psb.ugent.be/webtools/plantcare/html/, accessed on 5 October 2023). The distribution and composition of cis-elements in *ZmFTIP*s were visualized using TBtools v1.045 (https://github.com/CJ-Chen/TBtools-II/releases, accessed on 5 October 2023) [22].

### 2.5. RNA-Seq Database

The expression patterns of *ZmFTIP*s in maize under various conditions, including different organs, hormonal treatments, low phosphorus (LP), low nitrogen (LN), low zinc (LZn), heavy metal cadmium (Cd) exposure, heat stress, salt stress, drought, and cold stress, were analyzed using data from the Plant Public RNA-Seq database (https://plantrnadb.com, accessed on 5 October 2023) [23].

### 2.6. Synteny Analysis

The syntenic analysis between maize, including *Arabidopsis thaliana*, *Brachypodium distachyon*, *Glycine max*, *Hordeum vulgare*, *Sorghum bicolor* was used One Step McscanX-Super Fast in TBtools [22].

### 2.7. RNA Extraction, Reverse Transcription, and RT-PCR Analysis

Total RNA was extracted from the leaves and roots of maize using the Unizol Total RNA Extraction Reagent (RE703, Genesand, Beijing, China). Subsequently, the RNA was reverse-transcribed into cDNA using a reverse transcription kit (RK20429, ABclonal, Wuhan, China).

Total RNA extraction and cDNA synthesis were carried out according to the protocol described by Li et al. [24]. cDNA was subjected to qRT-PCR analysis using SYBR Green detection on a 7500 Real-Time PCR System (Thermo Fisher Scientific, Waltham, MA, USA). The qRT-PCR data were normalized using Zea mays *ZmTubulin* (*Zm00001eb390190*) as an internal control [25]. All qRT-PCR primer pairs are listed in Table S2.

## 3. Results

### 3.1. Identification of ZmFTIP Genes in Maize

In this study, the amino acid sequences of the C2 domain and the C-terminal phosphoribosyl transferase domain *FTIP1*, which are conserved in Arabidopsis, were used to identify analogs of the maize *FTIP1* protein through AMPS and Markov hidden model (MBE). A total of 27 FTIP proteins were found throughout the entire genome of maize. Based on their position on the chromosome, they were named *ZmFTIP1* to *ZmFTIP27* (Table S1). Characteristics of members of the *ZmFTIP* protein family, including identity (ID), chromosomal location, coding sequence (CDS) and amino acid sequence length, molecular weight (MW), isoelectric point (pI) of the encoded protein, and predicted subcellular localization are summarized (Table S1). The number of amino acids in ZmFTIP proteins varies from 166 to 1132, with molecular weights ranging from 18 to 124.4 kDa. The theoretical isoelectric points (pI) of all ZmFTIP members range from 4.75 to 10.28. The hydrophilicity coefficients of ZmFTIP family members vary between −0.327 and −0.102, while the instability indices of these proteins range from 25.55 to 62.92 (Table S1).

**Figure 1:**
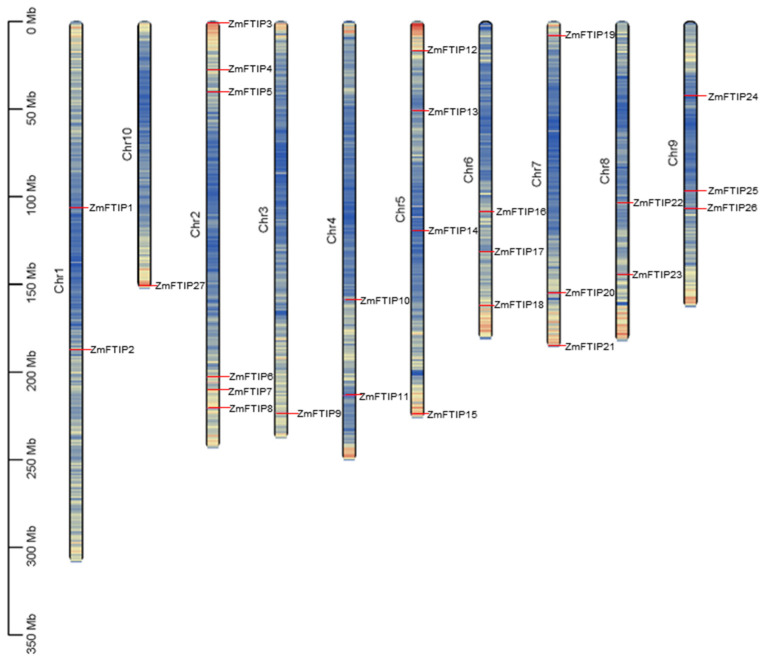
Chromosomal distribution of *FTIP* genes in the maize genome. The color bars represent the chromosomes, with chromosome numbers indicated on the left. *ZmFTIP* genes are marked to the right of the respective chromosomes. The scale bar on the left denotes the relative length of the chromosomes.

In order to understand the distribution of *ZmFTIP*s on the maize genome, we located *ZmFTIP*s at the exact location on the maize chromosome. However, the 27 *ZmFTIP*s are not evenly distributed across the 10 chromosomes of maize (Figure 1). One of them, *ZmFTIP1* and *ZmFTIP2*, is located on chromosome 1; *ZmFTIP3* to *ZmFTIP8* are located on chromosome 2; *ZmFTIP9* is located on chromosome 3 alone; *ZmFTIP10* and *ZmFTIP11* are located on chromosome 4; *ZmFTIP12*, *ZmFTIP13*, *ZmFTIP14*, and *ZmFTIP15* are located on chromosome 5; *ZmFTIP16*, *ZmFTIP17,* and *ZmFTIP18* are located on chromosome 6; *ZmFTIP19*, *ZmFTIP20*, and *ZmFTIP21* are located on chromosome 7; *ZmFTIP22* and *ZmFTIP23* are located on chromosome 8; *ZmFTIP24*, *ZmFTIP25*, and *ZmFTIP26* are located on chromosome 9; and *ZmFTIP27* is located on chromosome 10 (Figure 1). This suggests that the complexity and diversity of the *ZmFTIP*s gene family during evolution may be the result of multiple gene duplications and reconstructions. At the same time, the even distribution of members of the *ZmFTIP*s gene family on chromosomes may mean that they have certain independence in genetic regulation, each responsible for different physiological functions and metabolic pathways.

The subcellular localisation of *ZmFTIP*s was predicted using Cell-PLoc 2.0. *ZmFTIP1*, *ZmFTIP3*, *ZmFTIP5*, *ZmFTIP6*, *ZmFTIP7*, *ZmFTIP8*, *ZmFTIP9*, *ZmFTIP10*, *ZmFTIP17*, *ZmFTIP19*, *ZmFTIP22*, *ZmFTIP24*, *ZmFTIP25*, *ZmFTIP26* were located on the cytoplasm; *ZmFTIP2*, *ZmFTIP4*, *ZmFTIP7*, *ZmFTIP13*, *ZmFTIP14*, *ZmFTIP16*, *ZmFTIP18*, *ZmFTIP21*, *ZmFTIP23*, *ZmFTIP27* were located on the Cell membrane; *ZmFTIP1* and *ZmFTIP26* on the cytoplasm, or nucleus; *ZmFTIP5*, *ZmFTIP12*, *ZmFTIP15*, *ZmFTIP24* on the chloroplast, or nucleus; *ZmFTIP1* on the extra cell or mitochondrion (Table S1). The different subcellular localization of *ZmFTIP*s may be one of the important mechanisms for organisms to regulate protein functions, adapt to the environment, and maintain normal physiological activities.

**Figure 2:**
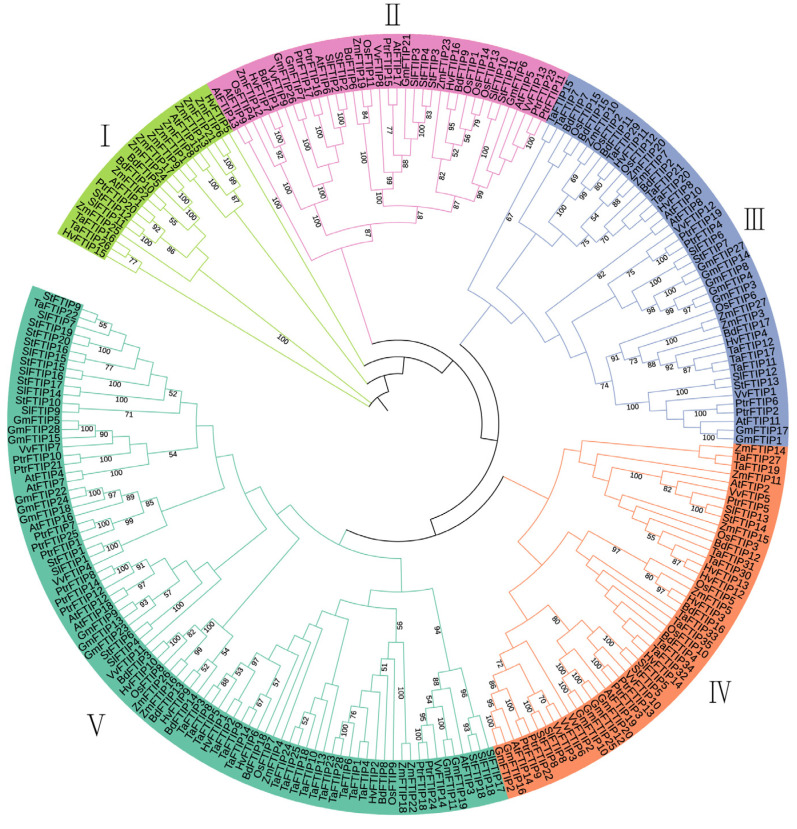
Phylogenetic analysis of *FTIP* proteins from *Zea mays* (Zm), *Glycine max* (Gm), Arabidopsis (At), rice (Os), *Brachypodium distachyon* (Bd), *Hordeum vulgare* (Hv), *Populus trichocarpa* (Ptr), *Solanum lycopersicum* (Sl), *Solanum tuberosum* (St), *Triticum aestivum* (Ta), and Vitis vinifera (Vv). The phylogenetic tree was reconstructed using MEGA 7.0 software by applying the maximum likelihood method. A bootstrap value of 1000 replicates was used to assess the robustness of the tree topology.

To analyze the evolutionary relationships of different FTIP proteins, maize, Arabidopsis, rice, soybean, *Brachypodium*, poplar, tomato, potato, barley, wheat, and grape were selected. Phylogenetic trees were reconstructed using the maximum likelihood method (Figure 2). The gene IDs, gene names, and protein sequences are detailed in Table S3. The maize FTIP family is divided into five subfamilies (Figure 2). Specifically, *ZmFTIP1*, *ZmFTIP2*, *ZmFTIP6*, *ZmFTIP8*, *ZmFTIP9*, *ZmFTIP13*, *ZmFTIP17*, *ZmFTIP21*, and *ZmFTIP25* belong to subfamily I; *ZmFTIP12*, *ZmFTIP19*, and *ZmFTIP23* are grouped into subfamily II; *ZmFTIP3*, *ZmFTIP7*, *ZmFTIP10*, *ZmFTIP20*, and *ZmFTIP27* are classified into subfamily III; *ZmFTIP5*, *ZmFTIP11*, *ZmFTIP14*, and *ZmFTIP15* are categorized into subfamily IV; and *ZmFTIP4*, *ZmFTIP16*, *ZmFTIP18*, *ZmFTIP22*, and *ZmFTIP26* are grouped into subfamily V (Figure 2). The distinct positions of *ZmFTIP* family members across various evolutionary branches suggest divergent genetic relationships among these genes, which may lead to functional differences.

**Figure 3:**
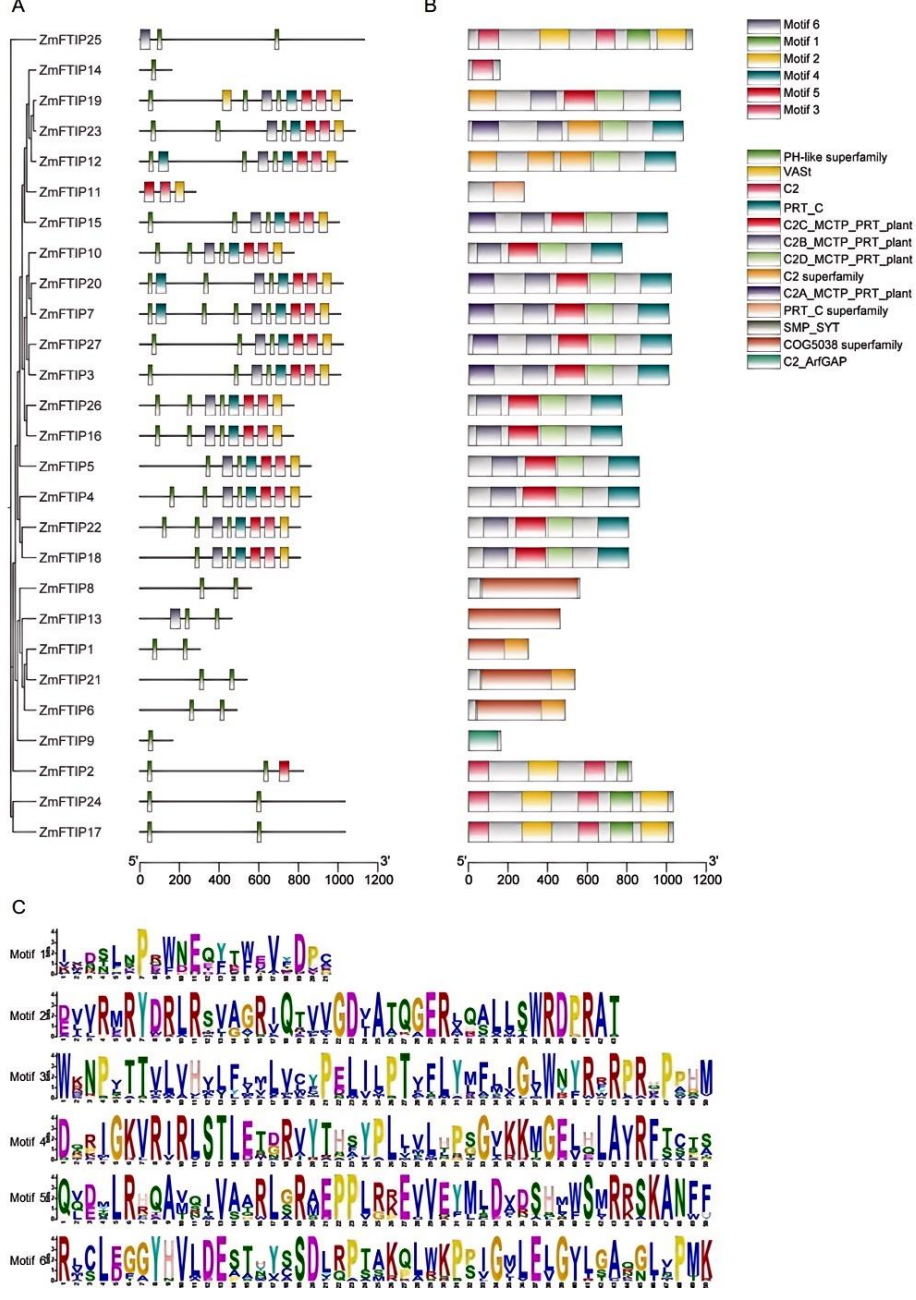
Motif and protein domain analysis of the ZmFTIP family: (**A**) motif composition, (**B**) protein domain structure, and (**C**) amino acid sequences of conserved motifs. The size of the letters in the motifs indicates the degree of conservation.

To further understand the functions of *ZmFTIP*s, conserved motifs and domains were analyzed. *ZmFTIP*s members mainly contain six conserved motifs (Figure 3A). The results showed that 27 *ZmFTIP*s contained one conserved motif, 19 *ZmFTIP*s contained two conserved motifs, and 15 *ZmFTIP*s contained six motifs (Figure 3A).

The structural characteristics of ZmFTIP proteins have also been investigated. All ZmFTIP proteins contain a C2C domain at their C-terminus and a C2 domain at their N-terminus (Figure 3B). Sequence alignment of *ZmFTIP*s reveals that the PRT-C domain and the C2 domain near the C-terminus are more conserved compared to the C2 domains located closer to the N-terminus (Figure 3B).

The amino acid sequences of each motif are characterized (Figure 3C). Additionally, the structure of ZmFTIP proteins is analyzed in detail. All ZmFTIP proteins contain a PRT-C domain at their C-terminus and three or four C2 domains at their N-terminus (Figure 3C).

### 3.2. Cis-Element Analysis of the ZmFTIP Genes in Maize

**Figure 4:**
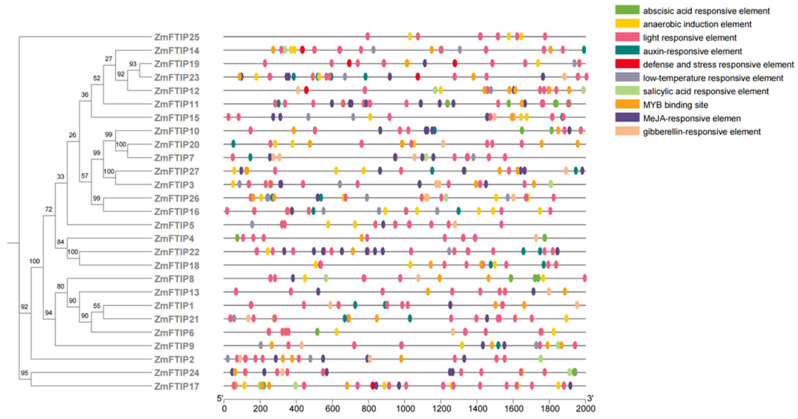
The cis-acting elements in the promoters of *ZmFTIP*s and their functions.

Promoter activity is essential for regulating gene function [26]. To elucidate the genetic function, metabolic network, and regulatory mechanisms of *ZmFTIP*, we analyzed the shared cis-elements within its promoter region. The 2000-base-pair upstream sequence of *ZmFTIP* was designated as the putative promoter. Using PlantCARE, we further investigated the distribution and functions of cis-elements in the *ZmFTIP* promoter region [27]. Our analysis revealed that the *ZmFTIP* promoter contains plant activation response elements, including auxin response elements, gibberellin response elements, abscisic acid response elements, and jasmonic acid (JA) response elements (Figure 4).

Auxin-responsive elements such as TGA elements (AACGAC) and AuxRR core (GGTCCAT) are present in 15 *ZmFTIP* genes (Figure 4). In addition, other plant hormone response elements were identified in the *ZmFTIP*s promoter region, including gibberellin (P-box; TATC box), JA (TGACG) motifs, abscisic acid (ABRE), and salicylic acid (TCA element). At the same time, we also analyzed that the promoter of the *ZmFTIP*s gene family mainly contains elements that respond to light, low temperature, and defense (Figure 4). This suggests that *ZmFTIP*s may play a potential role in plant stress defense and hormone response.

### 3.3. Analysis of the Expression of ZmFTIPs in Different Organs

**Figure 5:**
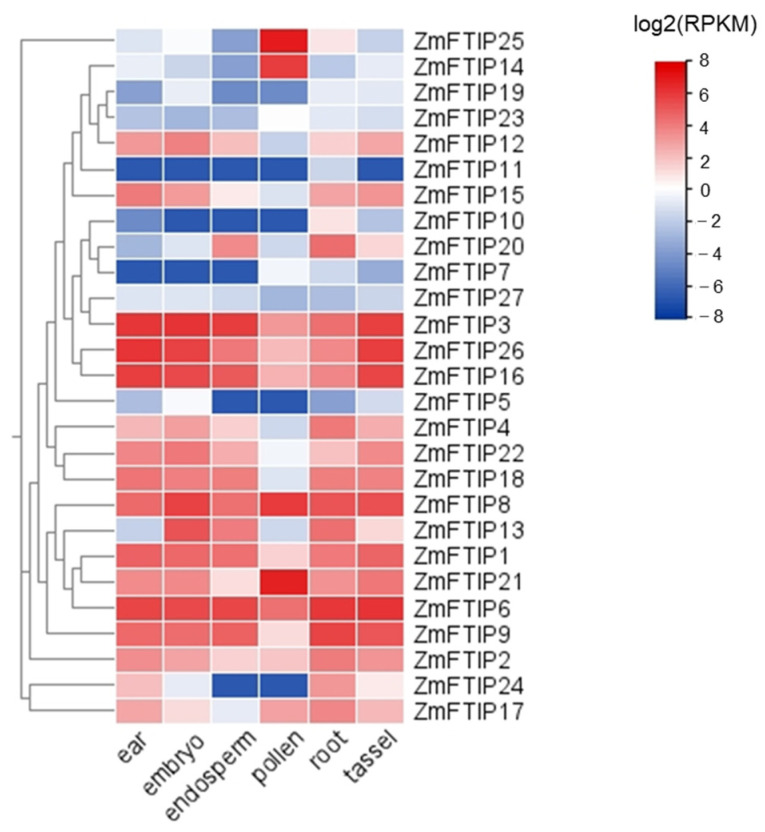
Expression patterns of *ZmFTIP*s in different maize organs. The relative expression levels of *ZmFTIP*s in the maize ear, embryo, endosperm, pollen, root, and tassel were obtained from published RNA-seq datasets. The color scale on the right represents the relative expression level (log2(FPKM)) of *ZmFTIP*s. RPKM: Reads per kilobase per million mapped reads. BioProject accession: PRJEB10406.

At first, we inquired about the expression of *ZmFTIP*s in different organs of maize in the Plant Public RNA-Seq database (https://plantrnadb.com, accessed on 5 October 2023) [23]. As shown in Figure 5, *ZmFTIP*s are expressed in the female ear, embryo, endosperm, pollen, root, and male ear of maize, and the expression levels of different *ZmFTIP*s genes are quite different. Among them, the expression levels of *ZmFTIP1*, *ZmFTIP2*, *ZmFTIP3*, *ZmFTIP4*, *ZmFTIP6*, *ZmFTIP8*, *ZmFTIP9*, *ZmFTIP12*, *ZmFTIP13*, *ZmFTIP16*, *ZmFTIP18*, *ZmFTIP21*, *ZmFTIP22*, and *ZmFTIP26* were higher than those of other members of *ZmFTIP*s gene family (Figure 5). Interestingly, the transcription level of *ZmFTIP14* and *ZmFTIP25* is high in pollen but low in other organs, indicating that these two genes may play a specific role in pollen development. The transcription level of *ZmFTIP*s is affected by sampling time and sampling environment, so further experiments are needed to analyze and prove the function of *ZmFTIP*s. Furthermore, research has demonstrated variations in the expression levels of *ZmFTIP*s across different maize organs, suggesting that individual members of the *ZmFTIP* family may fulfill distinct functions within specific tissue types.

### 3.4. Analysis of ZmFTIPs Expression in Response to Various Hormones

**Figure 6:**
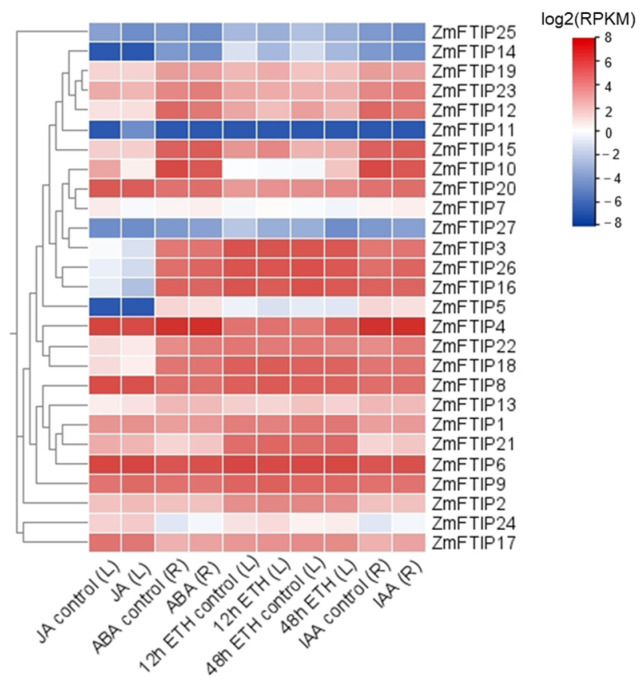
Expression patterns of *ZmFTIP*s in response to jasmonic acid (JA), abscisic acid (ABA), ethylene (ETH), and auxin (IAA) treatments. The relative expression levels of *ZmFTIP*s were extracted from published RNA-seq datasets. The color scale on the right represents the relative expression level [log2(RPKM)] of *ZmFTIP*s. RPKM: Reads per kilobase per million mapped reads. BioProject Accessions: JA, PRJNA380272; ABA, PRJNA546250; Auxin, PRJNA529334; Ethylene, PRJNA433298.

To investigate whether *ZmFTIP*s are regulated by hormones, the expression profiles of *ZmFTIP*s were systematically analyzed using data obtained from the Plant Public RNA-seq Database [23]. Expression profiles of 27 *ZmFTIP* genes were obtained (Figure 6). The expression of *ZmFTIP1*, *ZmFTIP17*, *ZmFTIP22*, and *ZmFTIP23* in maize roots was up-regulated by ABA treatment, while *ZmFTIP10* and *ZmFTIP12* were down-regulated by ABA treatment (Figure 6). JA treatment in maize leaves promoted the expression of *ZmFTIP1* but inhibited the expression of *ZmFTIP10* (Figure 6). The auxin analog IAA induced the up-regulation of *ZmFTIP1*, *ZmFTIP17*, *ZmFTIP22*, and *ZmFTIP23* genes in maize roots, while the down-regulation of *ZmFTIP10*, *ZmFTIP12*, and *ZmFTIP19* genes was induced by IAA treatment (Figure 6). Despite the absence of any identified cis-acting elements for cytokinin response in the promoter region of our *ZmFTIP*s, cytokinin treatment significantly modified the expression levels of *ZmFTIP*s (Figure 4 and Figure 6). After 12 h of cytokinin treatment in maize leaf cells, *ZmFTIP2*, *ZmFTIP15*, *ZmFTIP17*, and *ZmFTIP19* genes were up-regulated, and the expression of *ZmFTIP12* and *ZmFTIP23* genes were down-regulated; after 48 h of cytokinin treatment, *ZmFTIP12* and *ZmFTIP22* genes were up-regulated (Figure 6). In addition, we did not find relevant data on gibberellin and salicylic acid-treated maize in the Plant Public RNA-seq Database. Studies have demonstrated that plant hormones, including abscisic acid and ethylene, play a significant role in modulating plants’ responses to both biotic and abiotic stresses [28,29]. Given that the expression levels of certain *ZmFTIP*s family members are influenced by these hormones, it is plausible to hypothesize that *ZmFTIP*s may contribute to the regulation of maize’s adaptive responses to external stressors.

### 3.5. Comprehensive Analysis of ZmFTIPs Expression Profiles Under Diverse Stress Conditions

**Figure 7:**
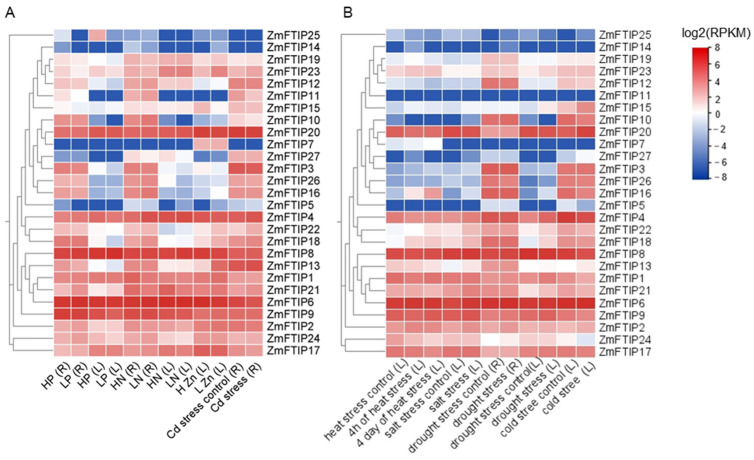
*ZmFTIP*s expression patterns in different stress. (**A**) *ZmFTIP*s expression patterns in low phosphorus (LP), low nitrogen (LN), low zinc (LZn), and cadmium (Cd) stress. (**B**) Expression patterns of *ZmFTIP*s in heat stress, salt stress, drought stress, and cold stress. The relative expression levels of *ZmFTIP*s were obtained from previously published RNA-seq datasets. The color scale on the right represents the relative expression level [log2(RPKM)] of *ZmFTIP*s. RPKM: Reads per kilobase/million. BioProject: LP, SRX793133; LN, SRX389606; LZn, PRJNA517889; Cd stress, PRJNA548845; heat stress, PRJNA396192; salt stress, PRJNA308155; drought stress, PRJNA284670; cold stress, PRJNA343268.

Furthermore, we identified cis-acting elements associated with defense and stress response within the promoter region of *ZmFTIP*s, leading us to hypothesize that *ZmFTIP*s may play a role in responding to nutrient deficiencies and other stress conditions. To investigate this hypothesis, we collected expression data for *ZmFTIP*s under low phosphorus (LP), low nitrogen (LN), low zinc (LZn), and heavy metal cadmium (Cd) stress (Figure 7A, Table S5).

Under LP stress, the expression levels of *ZmFTIP1*, *ZmFTIP2*, *ZmFTIP17*, *ZmFTIP21*, and *ZmFTIP24* in maize leaves were significantly up-regulated. Conversely, the expression levels of *ZmFTIP3*, *ZmFTIP13*, and *ZmFTIP16* were down-regulated. The expression levels of other *ZmFTIP*s remained unchanged. Additionally, in maize roots, the expression levels of *ZmFTIP9* and *ZmFTIP25* were also down-regulated (Figure 7A, Table S5).

In the roots of maize, LN stress up-regulated the expression of *ZmFTIP4*, *ZmFTIP10*, *ZmFTIP11*, *ZmFTIP12*, *ZmFTIP16*, *ZmFTIP17*, *ZmFTIP18*, *ZmFTIP22*, and *ZmFTIP24*, while it down-regulated the expression of *ZmFTIP2*, *ZmFTIP19*, and *ZmFTIP23*. In the leaves of maize, LN stress promoted the expression of *ZmFTIP4* but led to decreased expression of *ZmFTIP2*, *ZmFTIP21*, and *ZmFTIP27* (Figure 7A, Table S5).

In the leaves of maize, LZn stress significantly promoted the up-regulation of the expression levels of *ZmFTIP1*, *ZmFTIP13*, *ZmFTIP18*, *ZmFTIP22*, and *ZmFTIP23*. Conversely, the expression of ZmFTIP15 was notably down-regulated under LZn stress (Figure 7A).

Furthermore, we investigated the transcriptional responses of *ZmFTIP*s under heat, salt, drought, and cold stresses (Figure 7B, Table S5). After 4 h of heat stress, the expression levels of *ZmFTIP2*, *ZmFTIP16*, and *ZmFTIP18* were significantly upregulated, whereas those of *ZmFTIP4*, *ZmFTIP20*, and *ZmFTIP24* were markedly downregulated. No significant changes were observed in the expression levels of other *ZmFTIP*s (Figure 7B, Table S5). After 4 days of continuous heat stress, the expression levels of *ZmFTIP16* and *ZmFTIP18* remained upregulated, while those of *ZmFTIP4*, *ZmFTIP13*, *ZmFTIP17*, *ZmFTIP20*, *ZmFTIP21*, and *ZmFTIP24* were consistently downregulated (Figure 7B, Table S5).

Under salt stress, in maize, the expression levels of *ZmFTIP4*, *ZmFTIP9*, *ZmFTIP18*, *ZmFTIP21*, and *ZmFTIP22* were upregulated (Figure 7B, Table S5). In response to drought stress, the expression levels of *ZmFTIP13*, *ZmFTIP20*, and *ZmFTIP21* were upregulated, while that of *ZmFTIP26* was downregulated. Additionally, the expression levels of *ZmFTIP4*, *ZmFTIP15*, *ZmFTIP18*, *ZmFTIP21*, and *ZmFTIP22* in maize leaves were upregulated, with no significant changes in other *ZmFTIP*s (Figure 7B, Table S5). Under cold stress, the expression levels of *ZmFTIP2*, *ZmFTIP15*, and *ZmFTIP20* were upregulated, whereas those of *ZmFTIP1*, *ZmFTIP4*, *ZmFTIP8*, *ZmFTIP9*, *ZmFTIP10*, *ZmFTIP16*, *ZmFTIP17*, *ZmFTIP18*, *ZmFTIP21*, and *ZmFTIP22* were downregulated, with no significant changes in other *ZmFTIP*s (Figure 7B, Table S5).

The heavy metal cadmium (Cd) is not an essential element for plants. In recent years, due to excessive Cd content in soil, it has posed a serious threat to agricultural production safety [30,31]. It is important to highlight that our findings indicate varying transcription levels of the *ZmFTIP*s gene family members under cadmium (Cd) stress. Under cadmium stress, the expression levels of *ZmFTIP4*, *ZmFTIP18*, *ZmFTIP21*, *ZmFTIP22*, and *ZmFTIP23* were significantly up-regulated. Conversely, the expression levels of *ZmFTIP11*, *ZmFTIP16*, and *ZmFTIP17* were down-regulated under low cadmium stress conditions (Figure 7A, Table S5). The transcriptional levels of *ZmFTIP*s in response to cadmium (Cd) stress appear to indicate a potential mechanism for regulating Cd detoxification in maize. However, further research is required to elucidate the role of *ZmFTIP*s in this process, particularly through genetic overexpression and gene knockout studies.

### 3.6. RT-PCR Analysis of the Expression of ZmFTIPs

**Figure 8:**
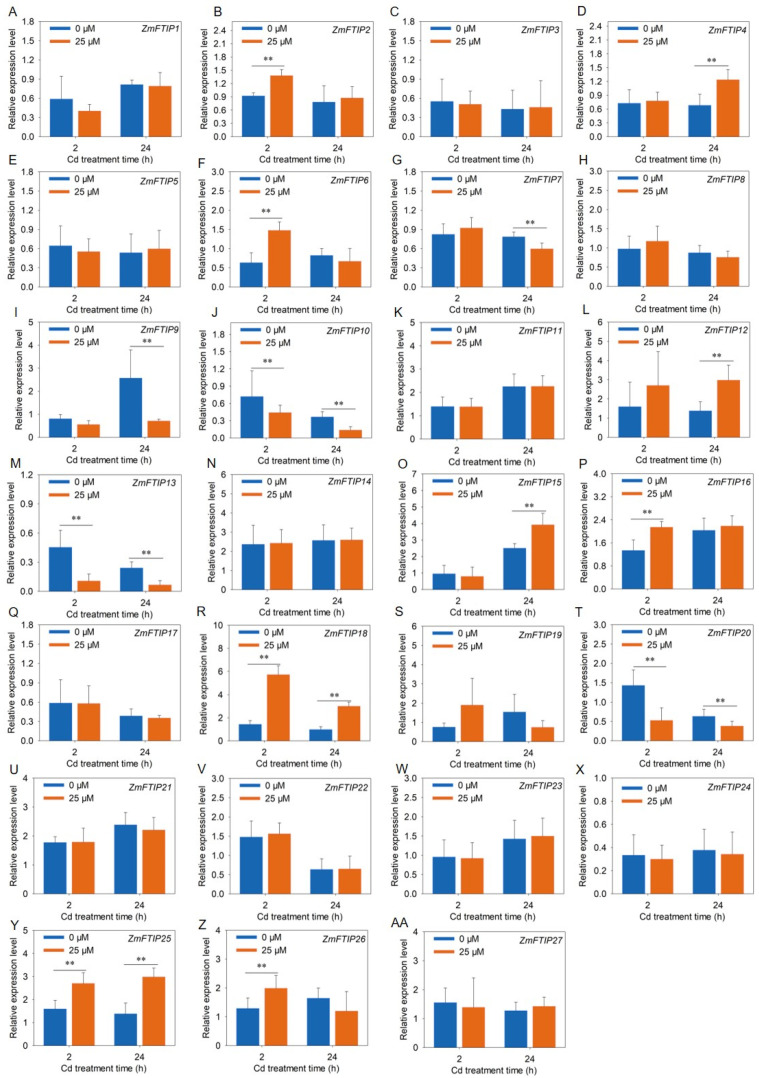
Analysis of expression level of *ZmFTIP* in maize root under cadmium stress. (**A**–**AA**) Responses of *ZmFTIP1*–*ZmFTIP27* to Cd stress in maize root. The relative expression levels of *ZmFTIP*s were normalized against *ZmTubulin* (*Zm00001eb390190*). Maize seedlings were grown in a hydroponic device for 10 days (d), then the seedlings were treated with 25 μM CdCl_2_, and samples were taken for measurement at 2 h (h) and 24 h, respectively. Data are presented as means ± SE (*n* = 4). Student’s *t*-test was employed to assess the significance of differences between 25 μM CdCl_2_ and 0 μM CdCl_2_ (**, *p* < 0.01).

**Figure 9:**
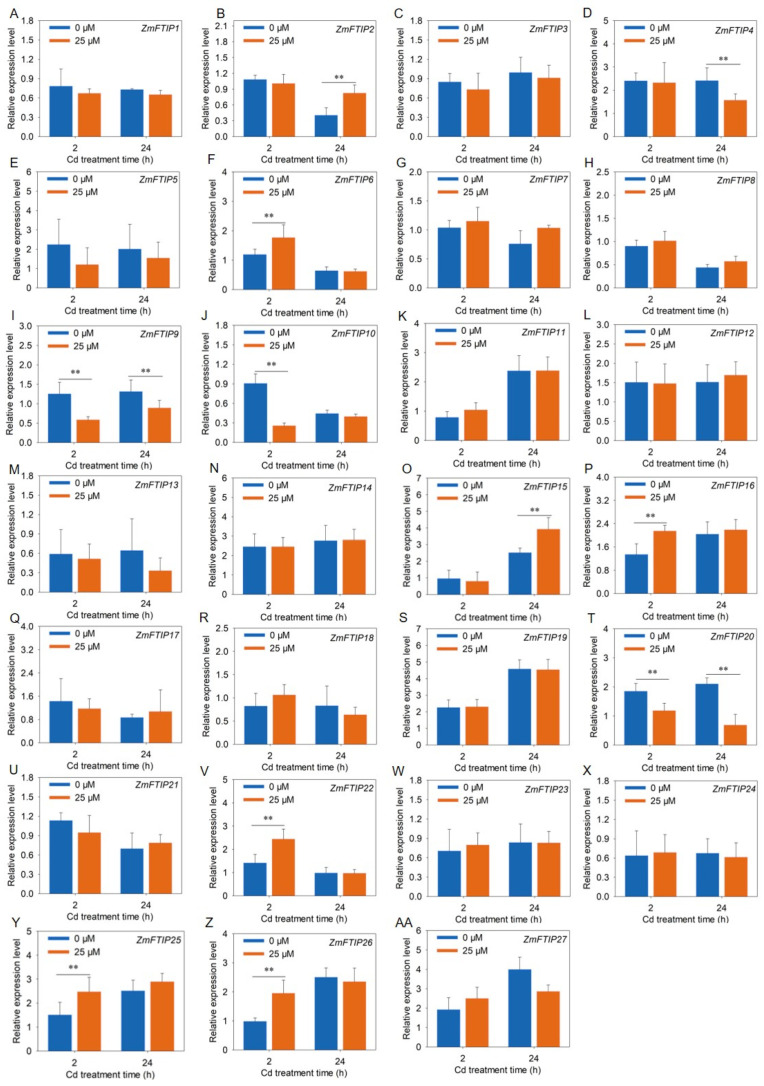
Analysis of the expression levels of *ZmFTIP*s in maize leaves under cadmium stress. (**A**–**AA**) Responses of *ZmFTIP1*–*ZmFTIP27* to cadmium stress in maize roots. The relative expression levels of *ZmFTIP*s were normalized against *ZmTubulin* (*Zm00001eb390190*). Maize seedlings were grown in a hydroponic device for 10 days (d), then the seedlings were treated with 25 μM CdCl_2_, and samples were taken for measurement at 2 h (h) and 24 h, respectively. Data are presented as means ± SE (*n* = 4). Student’s *t*-test was employed to assess the significance of differences between 25 μM CdCl_2_ and 0 μM CdCl_2_ (**, *p* < 0.01).

To further validate the responsiveness of *ZmFTIP*s to heavy metal cadmium stress, we employed RT-PCR to assess the relative expression levels of *ZmFTIP*s in both roots and leaves of maize under cadmium exposure (Figure 8 and Figure 9). As illustrated in Figure 8, the transcriptional activity of *ZmFTIP17*, *ZmFTIP18*, *ZmFTIP21*, and *ZmFTIP22* in roots markedly increased after 24 h of cadmium treatment, a trend that aligns closely with the RNA sequencing data from our prior study (Figure 7A, Table S5). Additionally, it was observed that after 2 h of cadmium exposure, the expression levels of *ZmFTIP6*, *ZmFTIP16*, *ZmFTIP25*, and *ZmFTIP26* in roots were notably elevated compared to untreated controls (Figure 8F,P,Y,Z), whereas the expression of *ZmFTIP10*, *ZmFTIP13*, and *ZmFTIP20* was significantly reduced (Figure 8J,M,T). To gain deeper insights into the expression dynamics of *ZmFTIP*s under prolonged cadmium exposure, we conducted an analysis of their relative expression after 24 h of cadmium treatment. The results indicated that in roots exposed to cadmium for 24 h, the expression levels of *ZmFTIP4*, *ZmFTIP12*, *ZmFTIP17*, *ZmFTIP18*, *ZmFTIP21*, *ZmFTIP22*, and *ZmFTIP25* were significantly upregulated compared to untreated samples, while those of *ZmFTIP9*, *ZmFTIP13*, and *ZmFTIP25* were significantly downregulated (Figure 8D,L,Q,R,U,V,Y). Notably, the expression of *ZmFTIP18* and *ZmFTIP25* in maize roots showed a significant increase at both 2 h and 24 h post-cadmium treatment (Figure 8R,Y), whereas the expression of *ZmFTIP13* was significantly decreased at these time points (Figure 8M). These findings suggest that the expression levels of *ZmFTIP13*, *ZmFTIP18*, and *ZmFTIP25* exhibit a relatively stable response to cadmium stress.

We utilized RT-PCR to evaluate the relative expression levels of *ZmFTIP*s in maize leaves exposed to cadmium. As shown in Figure 9, the transcriptional activity of *ZmFTIP17*, *ZmFTIP18*, *ZmFTIP21*, and *ZmFTIP22* in leaves significantly increased after 24 h of cadmium treatment, a trend that is consistent with RNA sequencing data (Figure 9R, Q,U,V). Furthermore, we observed that the expression levels of *ZmFTIP6*, *ZmFTIP16*, *ZmFTIP25*, and *ZmFTIP26* in leaves were markedly elevated after 2 h of cadmium exposure compared to untreated controls (Figure 9F,P,Y,Z), whereas the expression of *ZmFTIP10*, *ZmFTIP13*, and *ZmFTIP20* was significantly reduced (Figure 9J,M,T). To gain deeper insights into the expression dynamics of *ZmFTIP*s under prolonged cadmium exposure in leaf, we conducted an analysis of their relative expression after 24 h of cadmium treatment. The results indicated that in leaf exposed to cadmium for 24 h, the expression levels of *ZmFTIP4*, *ZmFTIP12*, *ZmFTIP17*, *ZmFTIP18*, *ZmFTIP21*, *ZmFTIP22*, and *ZmFTIP25* were significantly upregulated compared to untreated samples (Figure 9D,L,Q,R,U,V,Y), while those of *ZmFTIP9*, *ZmFTIP13*, and *ZmFTIP25* were significantly downregulated (Figure 9I,M,N). Notably, the expression of *ZmFTIP18* and *ZmFTIP25* in maize roots showed a significant increase at both 2 h and 24 h post-cadmium treatment, whereas the expression of *ZmFTIP13* was significantly decreased at these time points. These findings suggest that the expression levels of *ZmFTIP13*, *ZmFTIP18*, *ZmFTIP20*, and *ZmFTIP25* exhibit a relatively stable response to cadmium stress.

### 3.7. Synteny Analysis of ZmFTIPs Gene

**Figure 10:**
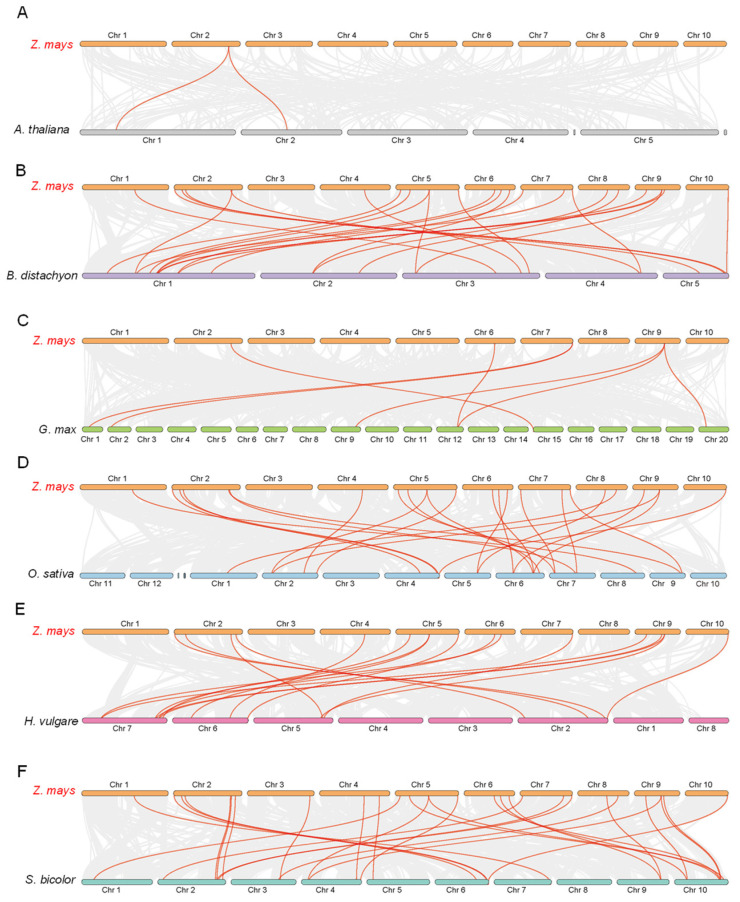
Syntenic analysis of *FTIP* genes between maize and six representative plant species is presented. The gray lines at the bottom represent collinear blocks within the maize and other plant genomes, while the red lines highlight the pairs of *FTIP* genes. The results of the syntenic analysis between maize, including *Arabidopsis thaliana*, *Brachypodium distachyon*, *Glycine max*, *Hordeum vulgare*, *Sorghum bicolor* (**A**–**F**).

To further explore the evolutionary relationship of *FTIP* proteins in crops and plants, we traced the collinear relationship between *ZmFTIP*s and homologs in other species. The number of homologous pairs among *ZmFTIP*s and among other species (Arabidopsis, Brachypodium, soybean, barley, sorghum) is 2, 24, 7, 24, 17, and 28, respectively. The results showed that there was a close genetic relationship between *FTIP*s and *ZmFTIP*s in Arabidopsis, *Brachypodium japonica*, soybean, barley, and sorghum (Figure 10).

*ZmFTIP6*, *ZmFTIP7*, and *ZmFTIP8* on chromosome 2 constitute the most homologous gene pairs compared to other species (Figure 10). Specifically, maize shares two homologous gene pairs with *Arabidopsis thaliana*, two with *Brachypodium distachyon*, one with *Glycine max*, two with *Oryza sativa*, two with *Hordeum vulgare*, and three with *Sorghum bicolor* (Figure 10A,B,D). For example, *ZmFTIP6* is collinear with *Arabidopsis thaliana AT1G20080* and *AT2G20990*, *ZFMTIP6* and *KQK17666* and *ZFMTIP7* and *KQK16235* are collinear, *ZmFTIP6* is collinear with *KRH51399* of soybean, *ZmFTIP6* is collinear with *Os09t0538800* of rice, *ZmFTIP7* is collinear with *Os07t0483500*, *ZmFTIP6* is collinear with *HORVU.MOREX.r2.5HG0415480* of barley, *ZmFTIP8* is collinear with *HORVU.MOREX.r2.2HG0098150* of barley, *ZmFTIP6* is collinear with *EER992650* in sorghum, *ZmFTIP7* is collinear with *EER99386*, and *ZmFTIP8* is collinear with *KXG36798*. These results suggest that these genes may play an important role in the FTIP gene family during evolution.

## 4. Discussion

In plants, the transport of nutrients, small molecules, and macromolecules between cells is mediated by plasmodesmata (PD), which are essential for regulating and enhancing intercellular communication [4]. Previous studies have demonstrated that the *MCTP* family, characterized by multiple C2 domains and transmembrane regions, plays a critical role in regulating macromolecule transport and cellular signaling [6,32]. Current research indicates that there are 16 members in Arabidopsis [11], 31 *MCTP*s identified in Gossypium hirsutum [33], and 13 members of the *MCTP* family in rice [12]. Maize, as an important grain crop with significant economic value [34,35], has limited biological information available for most of its *MCTP* members and those in other crop species. In this study, we identified 27 members of the *ZmFTIP*s distributed across 10 chromosomes (Table S1, Figure 1).

In recent years, researchers have discovered that *FTIP*s have a certain role in regulating plant flowering. *FT-interacting protein1* (*AtFTIP1*) is believed to affect the transportation of macromolecules [8]; among them, *AtFTIP1* and *AtFTIP6* play a certain regulatory role in controlling flowering time [8,12]. *OsFTIP1* plays a similar role in mediating rice flowering time under long days by affecting the transport of rice flowering locus T1, the rice counterpart of FLOWERINGLOCUST (FT), from companion cells to sieve elements [13]. Recently, it has been found that *ZmMCTP3* and *ZmMCTP10* in maize are closely related to *AtFTIP1*, which may participate in the floral transport pathway and affect the flowering process of plants, but their functions have not been verified by gene knockout or overexpression of *ZmMCTP3* and *ZmMCTP10* genes [17]. Therefore, whether *ZmFTIP*s are involved in the regulation of maize flowering remains to be further explored.

In a multi-species phylogenetic tree, genes within a subgroup typically exhibit similar functions [36]. The *AtFTIP* gene in *Arabidopsis thaliana* is divided into five subfamilies [10], the *OsFTIP* gene in rice is divided into six subfamilies [11], and the *FTIP* gene in upland cotton is divided into five subfamilies [33]. In this study, we made a phylogenetic analysis of the amino acid sequences of the *FTIP* family in maize and other species (Figure 2). The results showed that *FTIP* homologs from monocotyledonous rice, dicotyledonous *Arabidopsis thaliana*, and soybean were clustered in the same branch (Figure 2), indicating that the evolution of *FTIP* in these plants was close. *AtFTIP1* of *Arabidopsis thaliana* plays a role in regulating flowering. Phylogenetically, it shares similarities with *ZmFTIP1*, *ZmFTIP11*, *ZmFTIP18*, *ZmFTIP20*, *ZmFTIP22*, and *ZmFTIP25* (Figure 2). Nonetheless, additional research is warranted to ascertain whether it also plays a role in the regulation of flowering.

The majority of genes in plants belong to gene families. A gene family comprises a set of related genes that have evolved from a common ancestral gene through duplication and mutation events, resulting in genes with similar exon sequences [37]. Tandem replication is a form of gene replication that mainly occurs in the recombination region of chromosomes. In tandem replication, gene family members are usually closely arranged on the same chromosome, forming a gene cluster with similar sequence and function [37,38]. In maize, 27 *ZmFTIP* members are distributed on 10 chromosomes (Table S1, Figure 1). In order to know whether the *ZmFTIP* family is expanded by tandem and fragment duplication, the chromosome distribution and gene duplication of *ZmFTIP* in maize were analyzed (Figure 1 and Figure S1). Previous studies have confirmed the existence of gene tandem for *FTIP*s in Arabidopsis and rice [9,10,11]. Interestingly, a total of eight gene clusters and paired genes were identified on the chromosomes of 27 *ZmFTIP*s in maize (Figure S1), suggesting that the *ZmFTIP* gene family likely arose through tandem duplication and large-scale chromosomal duplication events. Most *ZmFTIP* genes may have originated from distinct ancestors, exhibiting high evolutionary diversity.

*MCTP* is a family of highly conserved proteins characterized by multiple C2 domains and transmembrane domains. Each protein contains three to four C2 domains at the N-terminus and one to four transmembrane domains at the C-terminus, functioning as key signaling molecules that mediate the transport of other regulatory factors in plant cells [8,12]. The C2 domain, one of the most common eukaryotic lipid-binding domains, serves as a docking module to target proteins to specific cell membranes through the formation of phospholipid complexes [5]. In membrane transport proteins, different C2 domains often exhibit distinct conserved sequences, suggesting that the multiple C2 domains within these proteins may function synergistically rather than being dedicated to specific cellular functions [6]. In maize, *ZmFTIP*s display typical features of *MCTP*s, possessing three to four C2 domains and one PRT-C domain (Figure 3B). Notably, the PRT-C domain and the C2 domain near the C-terminus are more conserved (Figure 3).

It has been shown that multiple C2 domain and transmembrane domain proteins (*MCTP*) family, the key regulatory factors of plant intercellular signal transduction, specifically act as ER-PM chains at plasmodesmata [8,12]. *MCTP*s are plasmodesmata proteins, which are inserted into the endoplasmic reticulum through their transmembrane regions, and their C2 domains are combined with the endoplasmic reticulum by interacting with anionic phospholipids [4,5,6]. The functional deletion mutant of *mctp3/mctp4* resulted in developmental defects in plants and impaired the function and composition of plasmodesmata. In contrast, the expression of *mctp4* in the yeast Dtether mutant restored the ER-PM connection [4,5,6]. OsFTIP1 is localized in the endoplasmic reticulum (ER), plasma membrane (PM), and cytoplasm in rice, playing a crucial role in the transport of RFT1 [8]. This study predicts the subcellular localization of *ZmFTIP* to the plasma membrane (PM), endoplasmic reticulum (ER), and cytoplasm (Table S1), suggesting that *ZmFTIP* may possess similar functions in these compartments. In addition, further research is required to elucidate the precise subcellular localization and functional roles of ZmFTIP proteins.

*ZmFTIP*s are expressed in the female ear, embryo, endosperm, pollen, root, and male ear of maize, and the expression levels of different *ZmFTIP* genes are quite different (Figure 5). The expression of *ZmFTIP3*, *ZmFTIP8*, and *ZmFTIP16* was the highest. The expression of *ZmFTIP7*, *ZmFTIP19*, and *ZmFTIP23* was the lowest in the *ZmFTIP* gene family (Figure 5).The differential expression patterns of *ZmFTIP*s across various organs highlight the diversity and specificity of their functional roles.

The distribution and types of cis-elements within promoters play a crucial role in determining the activity and function of genes. Auxin response elements such as TGA element (AACGAC) [39], AuxRR core element (GGTCCAT) [40] and gibberellin (P-box; TATCbox) and JA(TGACG) motifs [41]; Plant hormone response elements such as abscisic acid (ABRE) and salicylic acid (TCA) play an important role in gene response to various hormone regulation [42]. Interestingly, these cis-elements are common to most *ZmFTIP*s (Figure 4). At the same time, we found that the transcription level of some *ZmFTIP*s is regulated by hormone signals such as auxin, abscisic acid, and ethylene (Figure 6). These results indicate that the transcription factors related to plant hormone regulation may regulate the transcription of *ZmFTIP*s by binding homeopathic elements in the promoter region of *ZmFTIP*s.

In addition, the promoter of the *ZmFTIP*s gene family mainly contains cis-acting elements that respond to light, low temperature, and defense (Figure 4). These results suggest that the transcription level of *ZmFTIP*s may be regulated by abiotic stress. Previous studies have shown that some *FTIP* in *C. kwangsiensis* were up-regulated within 4 days under drought stress [43]. We found that the transcription level of some *ZmFTIP*s changed significantly under low phosphorus, low nitrogen, low zinc, cadmium stress, heat stress, salt stress, drought stress, and cold stress (Figure 7, Table S5). This indicated that *ZmFTIP*s might be involved in regulating the abiotic stress tolerance of maize to various nutrient stresses. Therefore, it is necessary to further predict and analyze the transcription factors that regulate the transcription of *ZmFTIP*s through bioinformatics and biochemical experiments.

Synteny analysis of *ZmFTIP*s and other *FTIP*-like proteins showed that we found more *FTIP* homologous pairs between rice and five monocot species, including maize, Brachypodium, sorghum, and barley, while there were seven homologous gene pairs between maize and soybean, and only seven homologous FTIP gene pairs between rice and Arabidopsis (Figure 10).

## 5. Conclusions

In this study, systematic identification and characterization of 27 *ZmFTIP* genes revealed their evolutionary divergence and functional diversification in maize. Phylogenetic classification into six clades highlighted monocot-specific conservation patterns, supported by synteny analysis indicating ancient evolutionary origins of *MCTP* genes. Promoter cis-regulatory element analysis uncovered abundant motifs linked to phytohormone signaling (ABA, JA, auxin) and stress adaptation. Expression profiling identified key stress-responsive candidates, with *ZmFTIP18* and *ZmFTIP25* demonstrating persistent upregulation under cadmium stress, suggesting specialized roles in heavy metal detoxification. Conversely, *ZmFTIP13* downregulation implies functional divergence in stress signaling pathways. These findings establish a foundational framework for exploring ZmFTIP-mediated intercellular communication mechanisms and provide valuable genetic resources for developing stress-resilient maize cultivars through biotechnological approaches.

## Data Availability

All data are reported in this manuscript.

## Supplementary Materials

The following supporting information can be downloaded at: https://www.mdpi.com/article/10.3390/genes16050539/s1, Figure S1: Syntenic analysis of FTIP genes in maize. In the genome-wide analysis of maize, collinear genes were connected by gray lines, while collinear FTIP genes were specifically highlighted using red lines; Table S1: Information of *ZmFTIP* family genes identified in maize genome; Table S2: Primer pairs used in this study. Table S3: The protein sequence of the plant FTIP family gene in 11 species has been analyzed; Table S4: FTIP family members screened using HMM models; Table S5: The Log(Fold Change) and *p*-values of maize FTIP genes under different stress.
