# Supplementary material for: Genome-Wide Identification and Phylogenetic Characterization of the FTIP Gene Family in Maize (Zea mays)
Source: Genes (Basel). 2025 Apr 30;16(5):539. doi: 10.3390/genes16050539 (PMC12111101; doi:10.3390/genes16050539)
Supplement: Supplementary file 1 [file genes-16-00539-s001.zip › genes-3577782-supplementary.pdf]

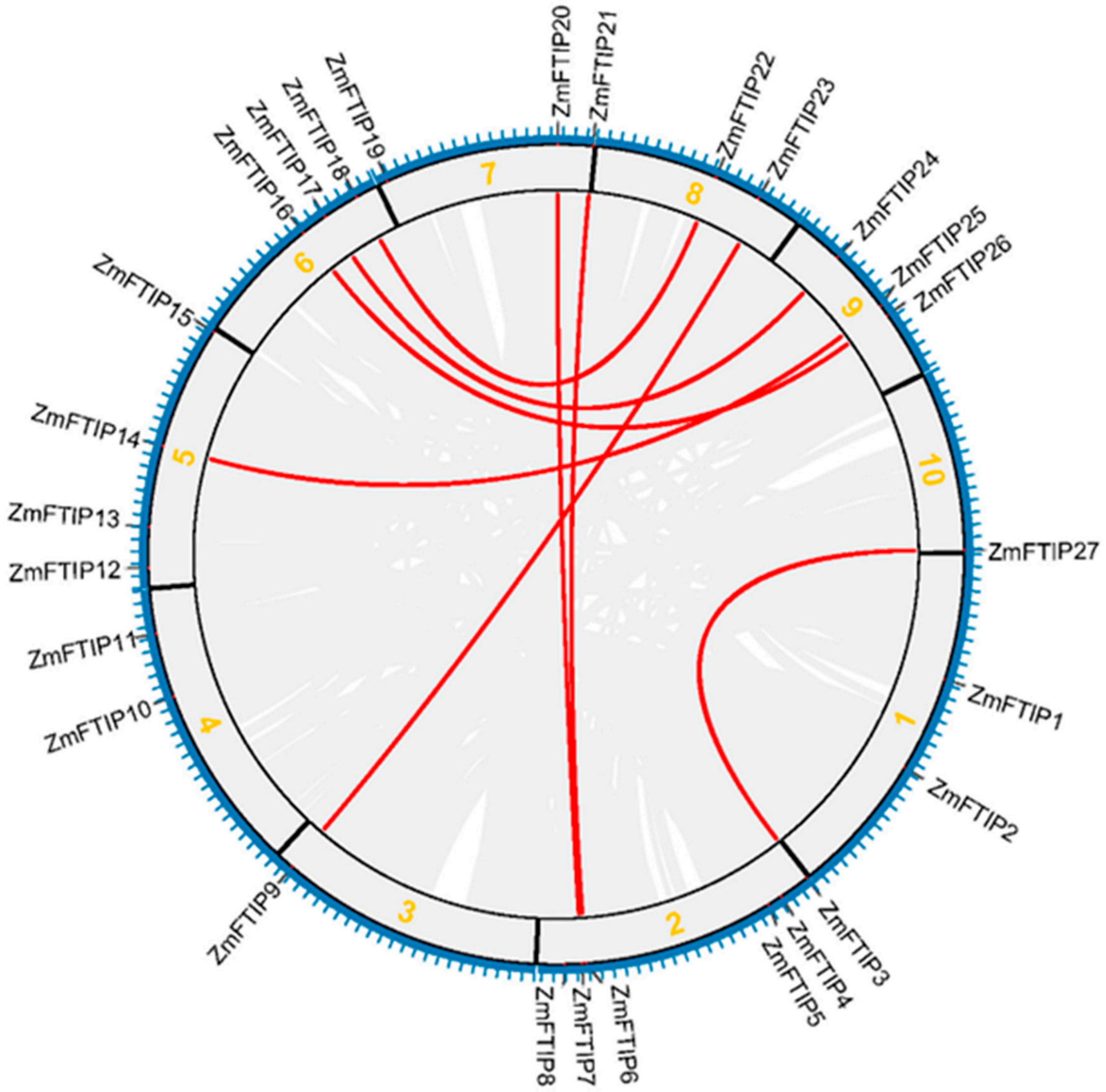

Figure S1 Syntenic analysis of FTIP genes in maize. In the genome-wide analysis of maize, collinear genes were connected by gray lines, while collinear FTIP genes were specifically highlighted using red lines.

Table S1. Information of ZmFTIP family genes identified in maize genome.

Gene name	Gene ID	Chromosome localization	gene model start	gene model end	Protein length (aa)	Protein MW (kDa)	pI	Instability index	Aliphatic index	GRAVY	Predicted subcellular localization
ZmFTIP1	Zm00001eb025070	chr1	106123715	106137375	304	34.63	6.64	37.2	82.24	-0.713	Cytoplasm. Nucleus.
ZmFTIP2	Zm00001eb033780	chr1	187380215	187393026	825	92.26	4.75	46.77	85.27	-0.299	Cell membrane.
ZmFTIP3	Zm00001eb065750	chr2	1123890	1128823	1013	114.93	9.17	35.66	85.76	-2.525	Cytoplasm.
ZmFTIP4	Zm00001eb076540	chr2	27650552	27654431	864	96.17	9.45	43.61	89.37	-0.133	Cell membrane.
ZmFTIP5	Zm00001eb079900	chr2	40139899	40142799	864	94.98	9.3	49.54	85.76	-0.102	Chloroplast. Cytoplasm.
ZmFTIP6	Zm00001eb103760	chr2	202766278	202773397	490	54.92	6.75	30.36	94.23	-0.251	Cytoplasm.
ZmFTIP7	Zm00001eb105000	chr2	206056207	206062009	1013	115.64	9.03	46.81	85.31	-0.302	Cell membrane.
ZmFTIP8	Zm00001eb110300	chr2	220549948	220582294	563	63.61	5.51	25.55	102.28	-0.107	Cytoplasm.
ZmFTIP9	Zm00001eb159470	chr3	223156631	223159138	166	18.7	5.95	42.57	114.91	-0.189	Cytoplasm.
ZmFTIP10	Zm00001eb186800	chr4	158700032	158702723	777	88.88	9.31	50.32	87.47	-0.145	Cytoplasm. Nucleus.
ZmFTIP11	Zm00001eb201170	chr4	213409659	213410583	282	31.95	10.28	55.08	98.9	0.169	Extracell. Mitochondrion.
ZmFTIP12	Zm00001eb218420	chr5	16488492	16492570	1047	114.34	8.98	46.43	82.4	-0.293	Chloroplast. Cytoplasm.
ZmFTIP13	Zm00001eb225520	chr5	50575813	50585822	464	53.16	9.04	39.07	94.6	-0.309	Cell membrane
ZmFTIP14	Zm00001eb235890	chr5	119270288	119270878	161	18	9.29	62.92	93.12	-0.196	Cell membrane. Cytoplasm.
ZmFTIP15	Zm00001eb258830	chr5	223950003	223954789	1006	111.48	9.42	47.77	81.34	-0.193	Chloroplast. Cytoplasm.

ZmFTIP16	Zm00001eb275770	chr6	108333659	108337797	775	89.47	9.19	42.51	91.73	-0.247	Cell membrane.
ZmFTIP17	Zm00001eb281080	chr6	131244147	131250381	1036	115.8	5.73	46.14	83.08	83.08	Cytoplasm.
ZmFTIP18	Zm00001eb289360	chr6	161894847	161897854	810	91.11	9.53	42.23	88.73	-0.108	Cell membrane.
ZmFTIP19	Zm00001eb300760	chr7	7751726	7755424	1071	116.91	6.53	49.28	80.6	-0.242	Cytoplasm.
ZmFTIP20	Zm00001eb321450	chr7	155259118	155265519	1025	116.12	8.87	43.09	83.64	-0.327	Cytoplasm. Nucleus.
ZmFTIP21	Zm00001eb332000	chr7	184918318	184933801	540	61.3	5.64	33.48	96.86	-0.26	Cell membrane.
ZmFTIP22	Zm00001eb348770	chr8	103822978	103825937	810	91.18	9.51	43.43	43.43	-0.103	Cytoplasm.
ZmFTIP23	Zm00001eb357370	chr8	143751859	143755656	1085	120.1	7.68	58.1	76.89	-0.313	Cell membrane.
ZmFTIP24	Zm00001eb380960	chr9	41822997	41833367	1035	116.11	5.69	41.12	82.37	-0.313	Chloroplast. Cytoplasm.
ZmFTIP25	Zm00001eb386570	chr9	96745753	96750888	1132	124.45	6.14	44.36	85.25	-0.2	Cytoplasm.
ZmFTIP26	Zm00001eb388260	chr9	106216663	106221088	776	89.17	9.17	41.45	92.89	-0.244	Cytoplasm. Nucleus.
ZmFTIP27	Zm00001eb434040	chr10	150840808	150843885	1026	116.35	9.18	36.12	86.86	-0.246	Cell membrane.

Table S2. Primer pairs used in this study.

Prime name	Primer sequence (5’-3’)	Purpose
ZmFTIP1-qF	GCAATCTCAATCCAGAGTGG	qRT-PCR
ZmFTIP1-qR	CATGTTTCCCAACCTGTTCC	qRT-PCR
ZmFTIP2-qF	TCTGCCGAGGAGACTTTCCTG	qRT-PCR
ZmFTIP2-qR	GGTACCAAGCGACAAGTCAT	qRT-PCR
ZmFTIP3-qF	CTACACTGTTTCCATCGGCA	qRT-PCR
ZmFTIP3-qR	CACTGTACTCCACTGTCAGC	qRT-PCR
ZmFTIP4-qF	AACCTTCTCCAGCAAGATCCA	qRT-PCR
ZmFTIP4-qR	GTGTACTGCTCGTTCCACTG	qRT-PCR
ZmFTIP5-qF	CGGCTGTCCTTATCATCGAG	qRT-PCR
ZmFTIP5-qR	GAAGTGCTCCAGGCTAAACC	qRT-PCR
ZmFTIP6-qF	AAGCTACTATCCAGATTGTGGA	qRT-PCR
ZmFTIP6-qR	TAGGTACTTCTAGTGTCTTCGG	qRT-PCR
ZmFTIP7-qF	GCGTTTCTCAAGAACAATAAGGTG	qRT-PCR
ZmFTIP7-qR	CTGCATGGTATACGTAGGCC	qRT-PCR

ZmFTIP8-qF	CCTCCATTTCTCAAAGTTGACAC	qRT-PCR
ZmFTIP8-qR	TTCCACCTTGCTCTTCTCCC	qRT-PCR
ZmFTIP9-qF	GCAAACAGAAATTGAAGACACG	qRT-PCR
ZmFTIP9-qR	CTGCATTACCCATTGTATCATCG	qRT-PCR
ZmFTIP10-qF	GAACGAGGACCACATGTTCG	qRT-PCR
ZmFTIP10-qR	GCACCAGATTAAACCATCTTGG	qRT-PCR
ZmFTIP11-qF	GCATTCAGGTGTTCATCGGT	qRT-PCR
ZmFTIP11-qR	ATCGTCCATCACAGTCACAG	qRT-PCR
ZmFTIP12-qF	ATCGTTTCATCTTCACTTCTCC	qRT-PCR
ZmFTIP12-qR	CCCACAATCAAACTCATCCA	qRT-PCR
ZmFTIP13-qF	CGCAGAACAAGAAGAACAGAGG	qRT-PCR
ZmFTIP13-qR	CTACCGAAACCAACAGCACC	qRT-PCR
ZmFTIP14-qF	ATTGCTCGATCTCTGTCCCT	qRT-PCR
ZmFTIP14-qR	CTGGAGCCGTTGAGGTAGAT	qRT-PCR
ZmFTIP15-qF	CGCTGATGAACTTCTTCCGA	qRT-PCR
ZmFTIP15-qR	GCCAAACTGACTTATCCACTG	qRT-PCR
ZmFTIP16-qF	TAAGCAGCCCAAAGATCCCT	qRT-PCR
ZmFTIP16-qR	CCATTACCAGTTTACCACCCT	qRT-PCR
ZmFTIP17-qF	GGATTGCGGAGAAATTCGTC	qRT-PCR
ZmFTIP17-qR	AGCAGCGTTTGATTTATCCTC	qRT-PCR
ZmFTIP18-qF	ACTCAAGAAGACTGGTGAGC	qRT-PCR
ZmFTIP18-qR	AACATATGTGAGTCCACATCGA	qRT-PCR
ZmFTIP19-qF	AAGGTGTACTACGTCGACGT	qRT-PCR
ZmFTIP19-qR	GTCATTACTTGTGCGTCTTCC	qRT-PCR
ZmFTIP20-qF	TGTCAAGCTGAATAATACTCCG	qRT-PCR
ZmFTIP20-qR	ACGTTGAAGTAGAGCTGCTC	qRT-PCR
ZmFTIP21-qF	CATGGCAAGGAAACTTTGGG	qRT-PCR
ZmFTIP21-qR	CAAAGAAACTATCTACACGAGCTC	qRT-PCR
ZmFTIP22-qF	ACACATTCCCTTCCTCCAGAC	qRT-PCR
ZmFTIP22-qR	ATGCCTATCACCATCAGAACC	qRT-PCR
ZmFTIP23-qF	CCTGATTATTCTCCATTTCGCT	qRT-PCR
ZmFTIP23-qR	GATTGAGATGTGACGATGCTG	qRT-PCR
ZmFTIP24-qF	GCTGAGCAAAGATCAGGGAC	qRT-PCR
ZmFTIP24-qR	TGACCATGCTTCTGTATACCC	qRT-PCR
ZmFTIP25-qF	GTTCTTTCTGTTGATGTCGAGTC	qRT-PCR
ZmFTIP25-qR	CTTGTCGAGCTTGTAGCTCG	qRT-PCR
ZmFTIP26-qF	TTTGTTGCAGAGGACAAGCG	qRT-PCR
ZmFTIP26-qR	TCAAGGAGTACTCCTCCGGA	qRT-PCR
ZmFTIP27-qF	GTCCTCGTTATCCTCCACAC	qRT-PCR
ZmFTIP27-qR	TACCTCACCCTTACAATCTCAG	qRT-PCR
qTUB-F	CTACCTCACGGCATCTGCTATGT	qRT-PCR
qTUB-R	GTCACACACACTCGACTTCACG	qRT-PCR
